# Predictors of Survival after Vaccination in a Pneumonic Plague Model

**DOI:** 10.3390/vaccines10020145

**Published:** 2022-01-19

**Authors:** Barry D. Moore, Clair Macleod, Lisa Henning, Robert Krile, Ying-Liang Chou, Thomas R. Laws, Wendy A. Butcher, Kristoffer M. Moore, Nicola J. Walker, Ethel Diane Williamson, Darrell R. Galloway

**Affiliations:** 1 Department of Pure and Applied Chemistry, University of Strathclyde, Glasgow G1 1XQ, UK; b.d.moore@strath.ac.uk (B.D.M.); clair.macleod@hotmail.co.uk (C.M.); 2 Battelle Biomedical Research Center, West Jefferson, OH 43162, USA; HenningL@battelle.org (L.H.); KrileR@battelle.org (R.K.); Chouyl@battelle.org (Y.-L.C.); 3 CBR Division, Dstl Porton Down, Salisbury SP4 0JQ, UK; trlaws@dstl.gov.uk (T.R.L.); wabutcher@dstl.gov.uk (W.A.B.); kmoore@dstl.gov.uk (K.M.M.); njwalker@dstl.gov.uk (N.J.W.); 4 Department of Pharmaceutics and Pharmaceutical Chemistry, University of Utah, Salt Lake City, UT 84112, USA; darrell.r.galloway@utah.edu

**Keywords:** plague, vaccine, formulation, immunity, correlates

## Abstract

**Background:** The need for an updated plague vaccine is highlighted by outbreaks in endemic regions together with the pandemic potential of this disease. There is no easily available, approved vaccine. **Methods:** Here we have used a murine model of pneumonic plague to examine the factors that maximise immunogenicity and contribute to survival following vaccination. We varied vaccine type, as either a genetic fusion of the F1 and V protein antigens or a mixture of these two recombinant antigens, as well as antigen dose-level and formulation in order to correlate immune response to survival. **Results:** Whilst there was interaction between each of the variables of vaccine type, dose level and formulation and these all contributed to survival, vaccine formulation in protein-coated microcrystals (PCMCs) was the key contributor in inducing antibody titres. From these data, we propose a cut-off in total serum antibody titre to the F1 and V proteins of 100 µg/mL and 200 µg/mL, respectively. At these thresholds, survival is predicted in this murine pneumonic model to be >90%. Within the total titre of antibody to the V antigen, the neutralising antibody component correlated with dose level and was enhanced when the V antigen in free form was formulated in PCMCs. Antibody titre to F1 was limited by fusion to V, but this was compensated for by PCMC formulation. **Conclusions:** These data will enable clinical assessment of this and other candidate plague vaccines that utilise the same vaccine antigens by identifying a target antibody titre from murine models, which will guide the evaluation of clinical titres as serological surrogate markers of efficacy.

## 1. Introduction

Whilst the world relies on effective vaccines for SARS-CoV-2 to end the world pandemic, another, much older biothreat still lingers. Bubonic plague is still endemic in parts of the world, predominantly existing in rodent populations from which it erupts periodically when conditions are favourable, resulting in several thousand fatal human cases globally each year [1]. Susceptible regions include Asia, Africa, Madagascar, South America and the Southwestern United States [1]. A live vaccine (EV76-NIIEG *Yersinia pestis*) is efficacious against endemic plague [2] but this vaccine is not approved for use in non-endemic regions where the risk/benefit ratio is higher and where it is considered to be too reactogenic.

Killed whole-cell vaccines for plague have been used since 1890 [3], with the most recent version from the Commonwealth Serum Laboratories [2]. Although effective vaccines, they are less effective in experimental models than is EV76 [4,5,6], and they are also are hazardous to produce.

Recently, more candidate vaccines have been pursued [7,8,9,10,11,12,13], but still there is no approved vaccine, although significant progress is being made. Awareness of the threat from plague was reawakened in 2017, with a particularly serious outbreak in Madagascar in which there were 2417 cases, a high proportion of them pneumonic, and 209 deaths, a case fatality rate of 8.6% [14].

Key to survival against plague is the induction of a vigorous immune response against the virulence factors produced by *Y. pestis* [15]. Although *Y. pestis* has evolved a myriad of virulence mechanisms to deter phagocytosis, promote host-cell adherence and suppress host immune responses [16,17,18,19], if just two of these are neutralised, people can survive infection. These two virulence mechanisms are mediated by the proteins termed Fraction 1 (F1) and Virulence (V) antigens, which, if used as immunogens, induce protective immunity [20].

Previously, we and others showed that the antibody titre to the F1 and V proteins, used as a mixture [20] or as a recombinant fusion protein [21], correlated with protection against bubonic and pneumonic plague in a range of animal models [22,23,24,25]. Subsequently, F1V vaccines were translated to the clinic in phase I/II trials [26,27,28]. Many different presentations of these antigens have been trialled with promising results [7], although the ability to scale-up formulation, enter economic production and achieve stability may also be as critical as novel formulation in vaccine success [29].

Recently, we showed that a simple modification to the conventional liquid alhydrogel formulation results in an efficacious vaccine in a murine bubonic model [30]. This formulation consists of biodegradable microcrystals comprising an amino acid matrix [31] into which the F1 and/or V proteins, or both, together with calcium phosphate are embedded. This protein-coated microcrystal (PCMC) formulation is a stable dry powder requiring no cold chain for storage and which can be reconstituted in alhydrogel prior to use by sub-cutaneous or intra-muscular injection in a two-dose regimen.

To facilitate the clinical development of the PCMC formulation, we have now carried out a murine efficacy study in which we varied a range of factors: the presentation of these two recombinant antigens in the form of a fusion protein or in a simple mixture (vaccine type); the dose level of the antigen administered; and the formulation used. We then investigated the relationship of vaccine type, dose and formulation to the protective efficacy against aerosolised challenge with *Y. pestis.*

Vaccines against biothreat agents such as *Y. pestis* can undergo conventional clinical trials for safety and immunogenicity, but phase III clinical trials for efficacy cannot be justified in the absence of a current outbreak for ethical reasons. In these situations, animal rule studies [32] can be carried out such that efficacy data in suitably qualified animal models [33] can be substituted for human efficacy data, as surrogate markers of efficacy. This process can be significantly enabled by the determination of an antibody titre cut-off in the animal model, above which survival of a pneumonic plague challenge can be predicted with a high degree of accuracy, an approach used successfully for other vaccines [34].

Here, we focused on the development of specific antibody titres to each antigen at two weeks after the booster dose (day 35) in order to correlate with subsequent protection, as it is important to have an early readout of titre, as well as indication of survivability from a clinical trial, and particularly, of course, in the event of a plague outbreak.

## 2. Materials and Methods

### 2.1. Animals

This study was performed at the Battelle Biomedical Research Center, USA; all procedures were approved by the IACUC. SPF 8–12-week-old Balb/c male and female mice (Charles River Laboratories, Wilmington, MA, USA) were allocated to treatment groups of 10 animals in a 50:50 ratio by gender. All mice were identified by Labstamp^®^ tail tattoos and implanted with a temperature transponder sub-cutaneously (s.c.; IPTT-300, BMDS, Seaford, DE, USA).

Protein antigens and formulation, immunisation and blood-sampling recombinant F1 and V [20] or F1-V [21] antigens were expressed from *Escherichia coli*, purified as previously described and supplied in PBS or ammonium acetate buffer for formulation.

For s.c. immunisation, individual F1 and V proteins or the F1-V fusion protein were prepared as calcium phosphate microcrystals using a published methodology [30]. Briefly, aqueous mixtures of proteins with sodium orthophosphate and glutamine (Gln) or histidine (His) were precipitated as calcium phosphate protein-coated microcrystals (CaP-PCMCs) through the addition of a 19-fold excess of isopropanol containing dissolved calcium chloride. The CaP-PCMCs were isolated by vacuum filtration and dried to a powder. Protein content and integrity were determined by ELISA and SDS-PAGE.

Mice were screened for pre-existing titres to the F1 antigen prior to entry to the study. Mice were immunised on day 0 and boosted on day 21, with the vaccine preparations shown in Table 1.

For immunisation with F1 and V or with F1-V in alhydrogel, volumes of each protein in PBS were added to a 20% *v*/*v* suspension of alhydrogel to deliver the immunising doses in 0.1 mL per mouse. For F1 and V or F1-V in the PCMC formulation, the mass of dry powder required to immunise 10 mice per group was pre-dispensed into vials, to which a suspension of 20% *v*/*v* alhydrogel in PBS was added to deliver the immunising doses in 0.1 mL per mouse.

Mice were blood-sampled every 14 days following immunisation by mandibular puncture. Serum was separated and stored at ≤−70 °C pending analysis.

### 2.2. Challenge

Mice were challenged on day 49 with *Y. pestis* CO92 in PBS + 0.01% gelatin with 9.7% α-α-trehalose (BSGT). The *Y. pestis* was checked for phenotype (>80% pigmented colonies on Congo Red agar), purity on solid agar culture and Gram stain.

Mice were challenged with a target-inhaled dose of 1.5 × 10^5^ colony-forming units (CFUs)/mouse *Y. pestis* CO92 within a BSL3 laboratory. *Y. pestis* CO92 suspensions in buffer were aerosolized with a six-jet Collison nebulizer and delivered to mice via a nose-only exposure chamber. Target-inhaled doses were delivered by controlling the aerosol concentration of *Y. pestis* and the exposure time and determined using Guyton’s formula: the group mean body weight of mice and the CFUs of *Y. pestis* in a known aerosol volume collected into buffer-containing impingers.

Challenged mice were subsequently monitored for 14 days, starting with checks every 8 h, followed by increasingly frequent checks with the onset of clinical signs. Mice were culled if they displayed clinical signs, set as the IACUC-approved humane end-point. All mice surviving at 14 days were culled. Spleens were excised and homogenates were plated onto CIN agar to recover *Y. pestis.*

### 2.3. Immunoassay

Specific IgG titre to the F1 and V antigens in blood serum was determined by ELISA as previously described [35] and presented as group mean ng/mL ± SD.

Neutralising antibody directed to the V antigen was assayed by competitive ELISA (20), modified such that murine immune sera were competed with biotinylated Mab7.3 for binding to V antigen (20). Samples were pooled and tested in duplicate. The data are reported as O.D._414nm_. These data were analysed using a three-parameter analysis of variance with Bonferroni’s post test using the software GraphPad PRISM V8.0.

### 2.4. Survival and Immunogenicity Statistical Analysis

Survival rates ± 95% confidence intervals were calculated for each treatment group, using exact binomial confidence intervals. One-sided Boschloo’s exact tests were performed to assess whether survival in each group was superior to group M.

The time-to-death (TTD) data combined with survival data were analysed by log-rank tests to determine differences in protection for treatment groups. In the log-rank test, TTD was censored at time from challenge to terminal sacrifice for animals that survived to the end of the study. If the overall log-rank test was significant, then pairwise log-rank tests were performed to determine which analysis groups were significantly different. Throughout, the Bonferroni–Holm adjustment for multiple comparisons was used to maintain an overall 0.05 level of significance. Both unadjusted and multiple comparison-adjusted outcomes are reported.

### 2.5. Statistical Modelling

The software JASP V13.1 was used for binary logistic regression and analysis of covariance (ANCOVA). Receiver operator curves (ROCs), analysis and associated graphs were generated using GraphPad PRISM V8.0. Dose-level and titre data were transformed by logarithm_10_ to fit a Gaussian distribution. The suitability of binary logistic regression was assessed using residual plots. The suitability of the ANCOVA was assessed using QQ plots and Levene’s tests for unequal variance. The “forward” method of model building was used for binary logistic regression. A three-parameter (serum dilution, vaccine formulation and type) analysis of variance was used to characterise the competitive ELISA dataset.

## 3. Results

### 3.1. Induction of Dose-Related Immune Responses

**Figure 1:**
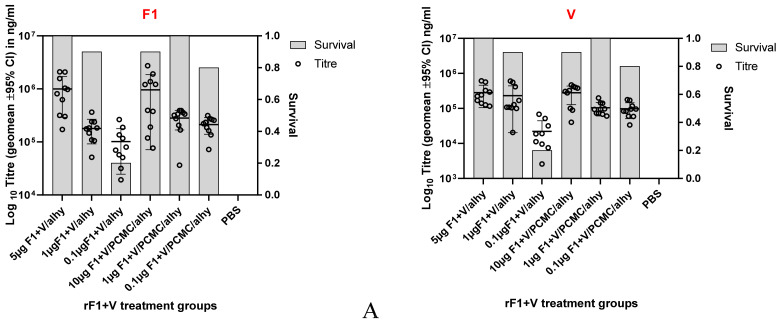

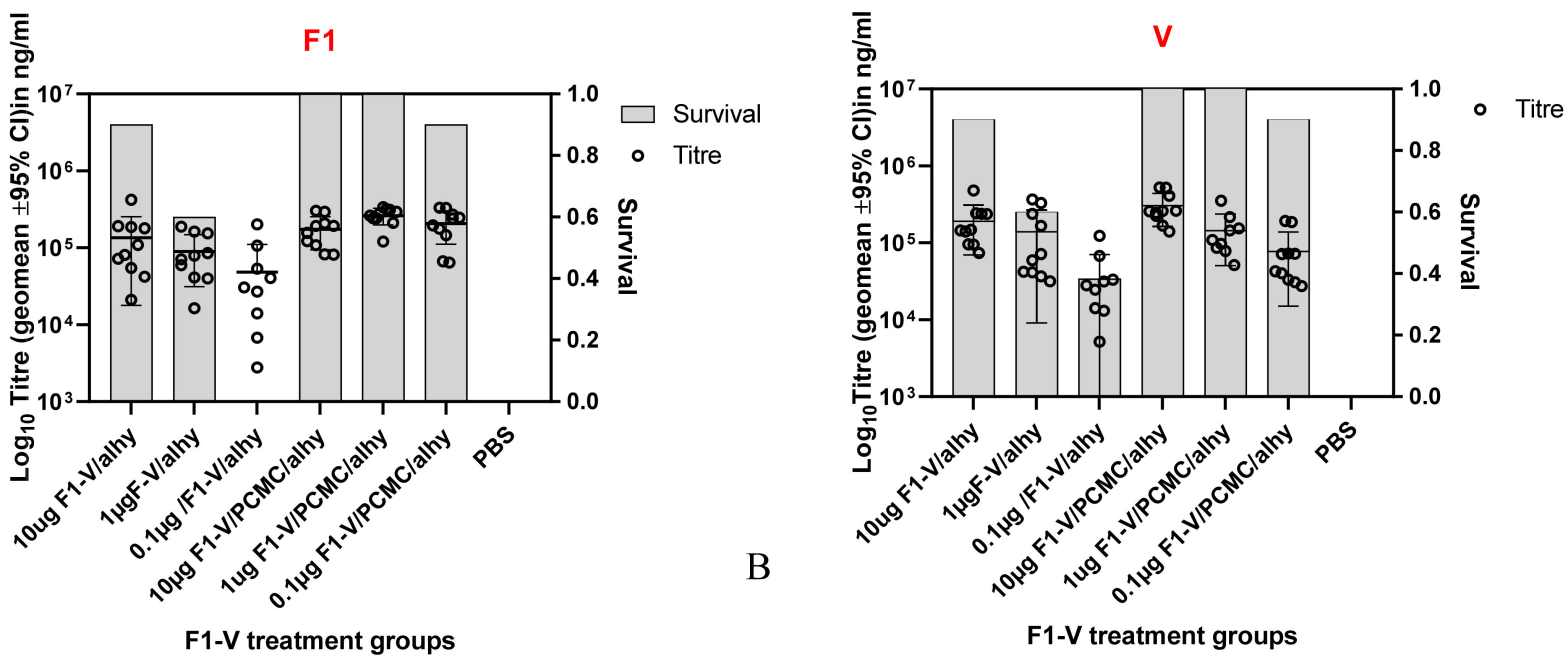
Heterogeneity of survival and antibody titres in immunised mice challenged with *Y. pestis*. The experiment consisted of 135 different immunisation conditions (*x*-axis), and survival after challenge with *Y. pestis* (bars, as a proportion, n = 10) was recorded. In addition, the antibody titres prior to challenge were determined (data points) in ng/mL for F1 antigen (left) and in ng/mL for V antigen (right). Panel (**A**) shows the response of groups immunised with F1 and V, whilst panel (**B**) shows the response of groups immunised with F1-V. All titres are shown as the geometric means ± SD.

Mice were immunised with either F1-V (fusion) or F1 and V (mixture)/alhydrogel or PCMC/alhydrogel (day 0, 21) (Table 1). Serum IgG to F1 was observed at day 14 for animals vaccinated with rF1 and rV or day 35 for animals vaccinated with F1-V. Across the treatment groups and irrespective of vaccine type, mice generally responded to immunisation in a dose-related manner, so that as antigen dose was titrated from 10 µg to 0.1 µg, antibody titre decreased (Figure 1). An exception to this was antibody titres to F1 when this antigen was presented as a mixture with V; in this case, a prozone effect was seen, where antibody titres to lower doses of F1 were enhanced by PCMC formulation. The day 35 antibody titres were also visually associated with the eventual survival data at 14 days p.i. on subsequent challenge of these treatment groups.

In this study, antibody titres which had developed by day 35 were selected for analysis. The reason for selecting this time point was twofold: at 14 days after the booster dose, the day 35 titre reflected the response to both the priming and boosting doses, and the use of this time point in a clinical trial would give an early readout of immunogenicity and a prediction of protection.

**Figure 2:**
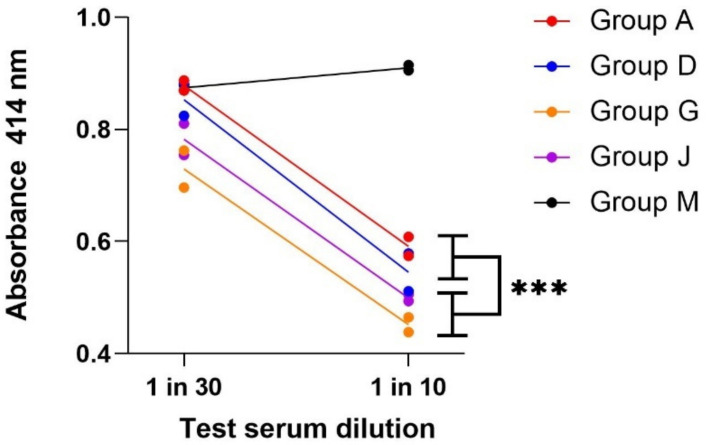
Competitive ELISA to determine the ability of day 35 sera, pooled by treatment group, to displace Mab7.3 from binding to V antigen. Pooled sera from each treatment group were assayed in duplicate in the dilution range 1:5 to 1:100 for their ability to displace 80 ng biotinylated Mab7.3 from V antigen, coated to solid phase. Pooled sera from group M provided the negative control. The OD_414nm_ was provided by streptavidin peroxidase detection of the biotinylated Mab7.3 and decreased as the test serum concentration increased. Group A: F1+V/alhydrogel; group D: F1+ V/PCMC/alhydrogel; group G: F1-V/alhydrogel; group J: F1-V/PCMC/alhydrogel; group M: PBS. These data were analysed using a three-parameter (serum dilution, vaccine formulation and type) analysis of variance with Bonferroni’s post test, using the software GraphPad PRISM V8.0. *** Group G sera were significantly more effective at displacing biotinylated Mab7.3 than are sera from Group A *p* < 0.001.

At this time point it was also important to determine the component of neutralising antibody (Nab) present (Figure 2). To do this, sera were pooled by treatment group and then assayed in duplicate by competitive ELISA. The data showed that immune sera contained Nab which competed with Mab7.3 for binding to the V antigen. Both vaccine types induced Nab to V antigen, with the F1-V being more effective than F1 and V when either was presented in alhydrogel. However, when the PCMC formulation was used, the F1-V presentation was not superior to F1 and V in induction of Nab to V.

### 3.2. Survival of Immunised Mice against Aerosol Challenge with Y. pestis

On day 49 mice were challenged by aerosol with an average of 7.99 × 10^4^ CFU *Y. pestis* CO92. The survival data by treatment group are shown in Table 1 and correlated with vaccine dose, so that, at the 10 µg antigen dose level, survival was 90–100%, dropping to 0–20% at the 0.1 µg dose level for both F1-V and F1+V/alhydrogel. In contrast, the formulation with F1+V or F1-V in PCMC/alhydrogel reversed this trend, so that, at the 0.1 µg dose level, the survival rate was 80–90%. Mice surviving at 14 days p.i. (day 63) had no culturable *Y. pestis* in their spleens, whereas mice which died prior to day 63 had culturable *Y. pestis.*

When median TTD per treatment group was compared to the negative control group M (Table 2), a significantly extended survival was found for all treatments, which significantly correlated with increasing dose level, as determined by pairwise log-rank test and Bonferroni–Holm adjusted log-rank test. The PCMC/alhydrogel-formulated F1+V and F1-V showed the greatest survival rates; 0.1 µg F1-V formulated in PCMC/alhydrogel (Group L) had the most extended TTD at 308.87 h compared with a mean of 61.85 h for the negative control (Group M).

### 3.3. Factors That Influence Survival in Immunised Mice Challenged with Y. pestis

**Figure 3:**
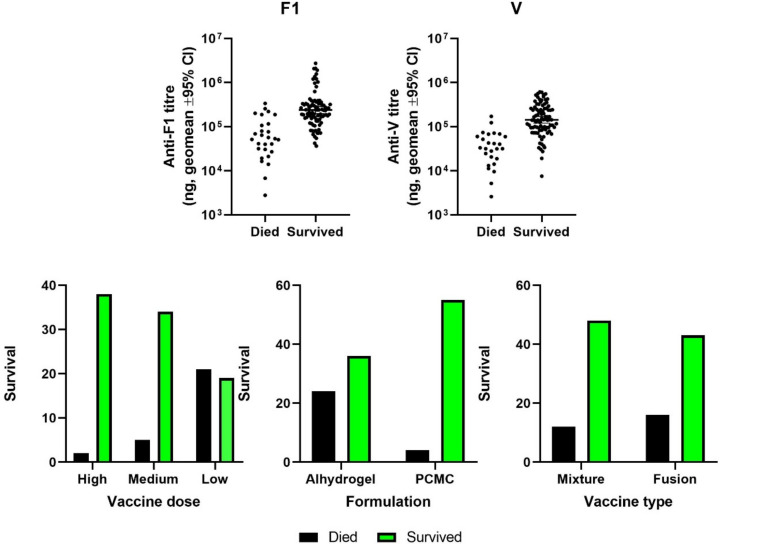
The factors that correlate with survival in mice immunised against *Y. pestis*. Top panel shows antibody titres raised against F1 (left) and V (right); lower panel shows the frequencies for animals in the study that died and survived when the vaccine was delivered at different concentrations (left), with different formulations (middle) or as a fusion or mixture of antigens (right). For F1, formulation in PCMC/alhydrogel interacted with dose level (*p* = 0.02) to influence survival, but both vaccine types were equally efficacious in this formulation. For V, antibody titre and dose level interacted to influence survival (*p* = 0.004), but both vaccine types were equally efficacious in PCMC/alhydrogel.

The immunisation conditions for each group of 10 mice were altered experimentally in three different ways: vaccine type, formulation and dose level. Here, we have linked the F1 and V antibody titres in individual mice to survival (Figure 3). In order to characterise which factors might relate to survival, two binary logistic regression models were generated, one for each antibody target (F1 or V). The “forward” method of statistical model building was used to identify all of the factors and interactions that correlate with survival (Table 3A).

For F1, formulation in PCMC/alhydrogel interacted with dose level (*p* = 0.02) to influence survival, but both vaccine types were equally efficacious in this formulation.

For V, antibody titre and dose level interacted to influence survival (*p* = 0.004), but both vaccine types were equally efficacious in PCMC/alhydrogel.

### 3.4. Factors That Influence the Antibody Response in Immunised Mice

**Figure 4:**
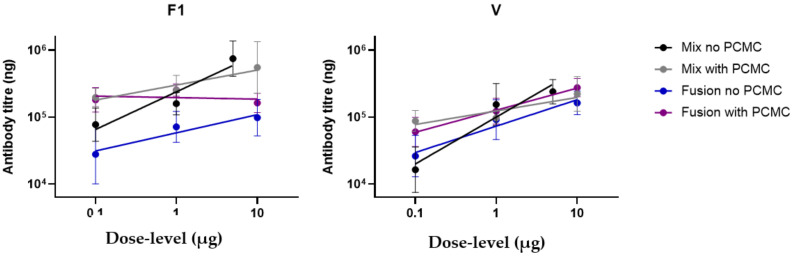
The antibody titres specific to antigen F1 (left) and V (right) of mice immunised against *Y. pestis*. The immunisations varied in three different ways: the dose level delivered (*x*-axis), formulation (with alhydrogel or with PCMC) and vaccine type (a mixture of F1 and V or a fusion).

ANCOVA was used to analyse how variation in antibody titres between treatment groups predicted survival (Figure 4). All the variables interacted to influence titres to F1 and V (Table 3B). However, the beneficial effect of the PCMC/alhydrogel formulation was generally in countering the effect of decreasing dose levels, particularly for F1-V, and preserving titres to both proteins.

### 3.5. Antibody Titres towards F1 and V Antigens Predict Survival with Challenge

**Figure 5:**
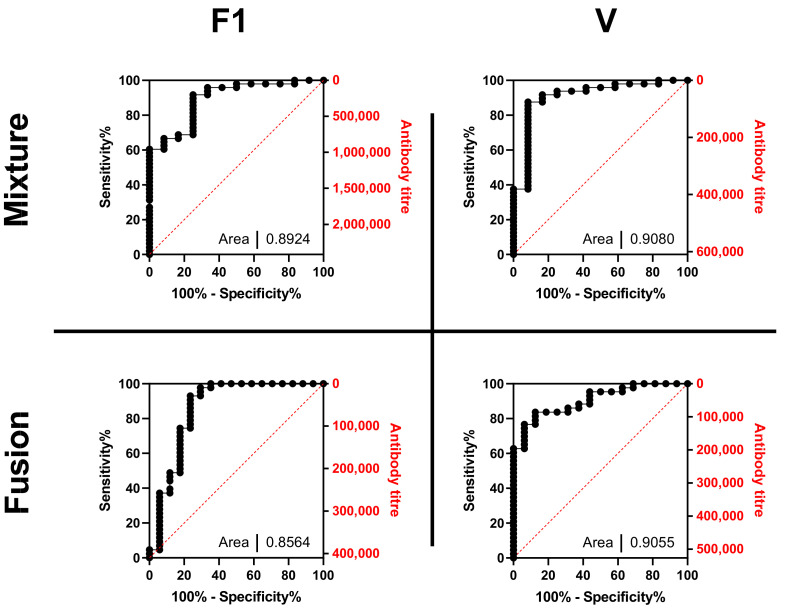
Receiver operator characteristic (ROC) curves of antibody titres in predicting survival after challenge with *Y. pestis* in immunised mice. These data include both titres specific to F1 (left) and V (right) and mice receiving a mixture of F1+V antigens (above) or the F1-V fusion protein (below). The ROC curves are shown in terms of titres (in ng/mL) and sensitivity.

Survival is the ultimate measure of protective immunity. However, as a binary measure it has little statistical power to allow for comparison of different treatments. To understand the nature of protection and the best surrogate marker of efficacy, ROC analysis [36] was performed for all the survival data generated, regardless of dose level and formulation (Figure 5). The vaccine types were analysed separately; the outputs indicated high AUC values with no major bias towards either sensitivity or specificity. However, this analysis also shows that F1 antigen performed less well in F1-V than in F1 and V, whilst there was little difference between vaccine types for V antigen. Since survival was enhanced when F1-V was presented in PCMC, the survival deficiency with F1-V in alhydrogel appears to be a limitation on titres to F1.

## 4. Discussion

Bridging the gap between R&D is a substantial hurdle in vaccine development, particularly where a phase III clinical trial of efficacy is impossible; here, animal efficacy data and the use of reliable surrogate markers of efficacy in blood draws in the clinic are critical. Whilst focussing here on the serological response, we are not suggesting that a cell-mediated response is not critically important in order to initiate an antibody response or in orchestrating a cellular response to pathogen challenge.

Here, we used an established murine model to analyse the antibody responses to F1 and V or F1-V and evaluate these as serological surrogate markers of efficacy. To achieve a gradient in efficacy, we titrated out the vaccine formulations and determined survival against a lethal aerosol challenge.

The statistical modelling of survival against experimental measurements revealed multiple correlating factors. For example, where both vaccine dose level and antibody were correlated to survival, we were left with the unanswered question of whether antibody just correlates to dose level and it is the dose level that promotes survival.

Here, we found from ROC analysis that the ability to predict survival from antibody titre to V antigen was greater in the mice receiving F1-V rather than F1 and V, whilst the converse was true for the F1 antigen. However, this differential was removed in the PCMC formulations of both vaccine types.

The data indicate that whist the response towards F1 may not be as significant in providing protection in this pneumonic model as the anti-V response, an induced titre to F1 is essential. Given that the evidence points to the antibody titres towards V correlating significantly with protection in this model, and NAb correlating with vaccine dose level, it is worth considering the utility of anti-V titres as a prognostic marker when F1V vaccines are escalated through NHP models to the clinic.

The observed ROC curves all had similar appearances. This is not surprising considering the known synergism between both titres. Here, the ROC curves showed high specificity and sensitivity. The AUC for V (across both vaccine types) was >0.9, considered ”excellent” for diagnostic purposes [36]. For V, it seems that titres above 200 µg/mL indicate a high likelihood of survival. In fact, no animal with a titre greater than 172 µg/mL died from infection, and we estimate that the likelihood of a vaccinated mouse with this threshold titre succumbing is less than 1 in 43 (<2.3%). We propose that in the forward development of the PCMC /alhydrogel formulation, an anti-V titre of 200 µg/mL be adopted as the threshold required for protective efficacy. F1 titres complement V titres and, in this context, an anti-F1 titre of 100 µg/mL predicts survival.

Based on this study and on our previous data, the PCMC/alhydrogel formulation is superior to alhydrogel for a number of reasons, including increased immunogenicity and efficacy and significantly increased stability [30], leading to a longer shelf life when stockpiled. The PCMC formulation entails a simple manufacturing process, which is readily scalable.

In order to extrapolate these findings to the clinic, it will be necessary to compare titres induced to the PCMC formulation of F1V in clinical trial volunteers with titres reported here and in planned NHP immunogenicity studies. Since the logarithm-transformed antibody response to immunisation in a diverse human cohort is likely to adopt a normal distribution, the aim would be to derive serum from low, medium and high-responder cohorts for analyses as proposed previously [37,38,39,40]. Alternatively, the ability to generate NAb towards the V antigen in all species is a precise metric that can be determined ex vivo and may be used to predict whether immunisation in the clinic will lead to protective efficacy.

## 5. Conclusions

These data underline the fact that immunity to both the F1 and V antigens is essential for vaccine efficacy. In the murine pneumonic model, serum titres of 200 µg/mL (V) and 100 µg/mL (F1) predict survival. Finally, the fusion of F1 to V limited the anti-F1 response, but microcrystal formulation overcame this to enhance survival.

## Figures and Tables

**Table 1:** Treatment groups and survival data.

Group ID	Number of Mice	Vaccine Type and Formulation	Vaccine Concentration: Dose Level of Each Protein Antigen (F1 and V) or of the Fusion Protein in µg/Mouse	Prime	Boost	Died	% Survival	Culturable *Y. pestis* in Spleens Post Mortem
				(Day 0)	(Day 21)			
A	10	rF1 + rV/alhydrogel	5 *	SC	SC	0	100	0
B	10		1			1	90	1
C	10		0.1			8	20	8
D	10	rF1 + rV (PCMC/alhydrogel)	10	SC	SC	1	90	1
E	10		1			0	100	0
F	10		0.1			2	80	2
G	10	rF1V fusion/alhydrogel	10	SC	SC	1	90	2
H	10		1			4	60	4
I	10		0.1			10	0	9
J	10	rF1V fusion (PCMC/alhydrogel)	10	SC	SC	0	100	0
K	10		1			0	100	0
L	10		0.1			1	90	1
M	15	PBS	NA	SC	SC	15	0	15

Where mice were dosed with a mixture of F1 and V, they received equal amounts of each protein in µg, as stated in the vaccine concentration column. Where mice were dosed with the fusion protein, they received the stated amount in µg. * Group A received 5 µg of each protein for comparison with Group G.

**Table 2:** Time-to-death data per treatment group.

Group	No. Dead/No. in Group	Kaplan–Meier Median Time to Death in Hours (95% Confidence Interval)	Overall Log-Rank Test *p*-Value	Pairwise Log-Rank Test *p*-Value			Bonferroni–Holm Adjusted Log-Rank Test *p*-Value		
				B	C	M	B	C	M
A	0/10	NA	<0.0001 *	0.3173	0.0002 *	<0.0001 *	1.0000	0.0143 *	<0.0001 *
B	1/10	--- (120.35, ---)		-	0.0012 *	<0.0001 *	-	0.0661	<0.0001 *
C	8/10	109.07 (91.18, 235.78)		-		<0.0001 *	-	-	0.0004 *
M (control)	15/15	61.85 (60.60, 66.67)		-	-	-	-	-	-
				E	F	M	E	F	M
D	1/10	--- (96.47, ---)	<0.0001 *	0.3173	0.5842	<0.0001 *	1.0000	1.0000	<0.0001 *
E	0/10	NA		-	0.1464	<0.0001 *	-	1.0000	<0.0001 *
F	2/10	--- (107.78, ---)		-	-	<0.0001 *	-	-	<0.0001 *
M (control)	15/15	61.85 (60.60, 66.67)		-	-	-	-	-	-
				H	I	M	H	I	M
G	1/10	--- (96.50, ---)	<0.0001 *	0.1469	<0.0001 *	<0.0001 *	1.0000	0.0017 *	<0.0001 *
H	4/10	--- (91.53, ---)		-	0.0005 *	<0.0001 *	-	0.0308 *	0.0001 *
I	10/10	96.34 (60.82, 108.27)		-	-	0.0001 *	-	-	0.0087 *
M (control)	15/15	61.85 (60.60, 66.67)		-	-	-	-	-	-
				K	L	M	K	L	M
J	0/10	NA	<0.0001 *	1.0000	0.3173	<0.0001 *	1.0000	1.0000	<0.0001 *
K	0/10	NA		-	0.3173	<0.0001 *	-	1.0000	<0.0001 *
L	1/10	--- (308.87, ---)		-	-	<0.0001 *	-	-	<0.0001 *
M (control)	15/15	61.85 (60.60, 66.67)		-	-	-	-	-	-

---, Median time-to-death and upper 95% confidence interval could not be estimated; *, overall group effect or difference between groups for time-to-death was significant at the 0.05 level. Group A: 5 µg F1+V/alhydrogel; B: 1 µg F1 +V/alhydrogel; C: 0.1 µg F1+V/alhydrogel; D: 10 µg F1 + V/PCMC/alhydrogel; E: 1 µg F1 +V/PCMC/alhydrogel; F: 0.1 µg F1 +V/PCMC/alhydrogel; G: 10 µg F1-V/alhydrogel; H: 1 µg F1-V/alhydrogel; I: 0.1 µg F1-V/alhydrogel; J: 10 µg F1-V/PCMC/alhydrogel; K: 1 µg F1-V/PCMC/alhydrogel; L: 0.1 µg F1-V/PCMC/alhydrogel; M: PBS.

**Table 3:** Summary of the results of statistical analysis.

(A) Factors Significantly Influencing Survival Determined by Binary Logistic Regression		
F1 or V	Factors	Significance
F1	Higher titres	*p* < 0.001
	PCMC and dose level	Significant interaction *p* = 0.02
	Vaccine type (F1 and V versus F1-V)	Not significant
V	Higher titres	*p* = 0.001
	PCMC/alhydrogel	*p* < 0.001
	Higher dose level	*p* = 0.005
	Titre dose level	Significant interaction *p* = 0.004
	Vaccine type	Not significant
**(B) Factors significantly Influencing Survival Determined by ANCOVA**		
**F1 or V**	**Interacting Variables**	**Significance**
F1	Effect of vaccine type altered by formulation (PCMC/alhydrogel)	*p* < 0.001
	Effect of vaccine type altered by dose level	*p* = 0.001
	Effect of formulation (with PCMC and without) altered by dose level	*p* < 0.001
V	Effect of dose level on titre	*p* < 0.001
	Effect of PCMC/alhydrogel on titre	*p* = 0.007
	Dose level and formulation in PCMC/alhydrogel	*p* < 0.001
	Titre and dose level and formulation in PCMC/alhydrogel	Significant interaction *p* = 0.006

## Data Availability

All data are contained within this article. Content includes material subject to © Crown copyright (2021), Dstl. This material is licensed un-der the terms of the Open Government Licence except where otherwise stated. To view this li-cence, visit http://www.nationalarchives.gov.uk/doc/open-government-licence/version/3 (accessed on 12 July 2021).
